# Silencing the Girdin gene enhances radio-sensitivity of hepatocellular carcinoma via suppression of glycolytic metabolism

**DOI:** 10.1186/s13046-017-0580-7

**Published:** 2017-08-15

**Authors:** Li Yu, Yifan Sun, Jingjing Li, Yan Wang, Yuxing Zhu, Yong Shi, Xiaojun Fan, Jianda Zhou, Ying Bao, Jie Xiao, Ke Cao, Peiguo Cao

**Affiliations:** 1 Department of Oncology, Third Xiangya Hospital, Central South University, Tongzipo Road, Changsha City, Hunan Province 410013 China; 2 Department of Clinical Laboratory, Affiliated Liutie Central Hospital of Guangxi Medical University, Fei-e Road, LiuZhou, Guangxi 545007 China; 3 Department of Plastic and Reconstructive Surgery, Third Xiangya Hospital of Central South University, Changsha, 410013 China; 4 Department of Scientific Research, Third Xiangya Hospital of Central South University, Changsha, 410013 China

**Keywords:** Girdin, Primary hepatocellular carcinoma, Glycolysis, Radio-sensitivity

## Abstract

**Background:**

Radiotherapy has been used increasingly to treat primary hepatocellular carcinoma. Clinically, the main cause of radiotherapy failure is cellular radioresistance, conferred via glycolytic metabolism. Our previous study demonstrated that Girdin is upregulated in primary hepatocellular carcinoma and promotes the invasion and metastasis of tumor cells. However, whether Girdin underlies the radio-sensitivity of hepatocellular carcinoma remains unclear.

**Methods:**

A short hairpin RNA (shRNA) was used to silence *CCDC88A* (encoding Girdin), and real-time PCR was performed to determine *CCDC88A* mRNA expression. Then, cell proliferation, colony formation, flow cytometric, scratch, and transwell assays were to examine the influence of Girdin silencing on cellular radiosensitivity. Glycolysis assays were conducted to exam cell glycolysis process. Western blotting was performed to explore the signaling pathway downstream of Girdin. Finally, animal experiments were performed to demonstrate the effect of *CCDC88A* silencing on the radiosensitivity of hepatoma in vivo.

**Results:**

shRNA-induced Girdin silencing suppressed glycolysis and enhanced the radio-sensitivity of hepatic cell lines, HepG2 and Huh-7. Furthermore, silencing of Girdin inhibited the PI3K/AKT/HIF-1α signaling pathway, which is a central regulator of glycolysis.

**Conclusion:**

Girdin can regulate glycolysis in hepatocellular carcinoma cells through the PI3K/AKT/HIF-1α signaling pathway, which decreases the sensitivity of tumor cells to radiotherapy.

**Electronic supplementary material:**

The online version of this article (doi:10.1186/s13046-017-0580-7) contains supplementary material, which is available to authorized users.

## Background

**Fig. 1:**
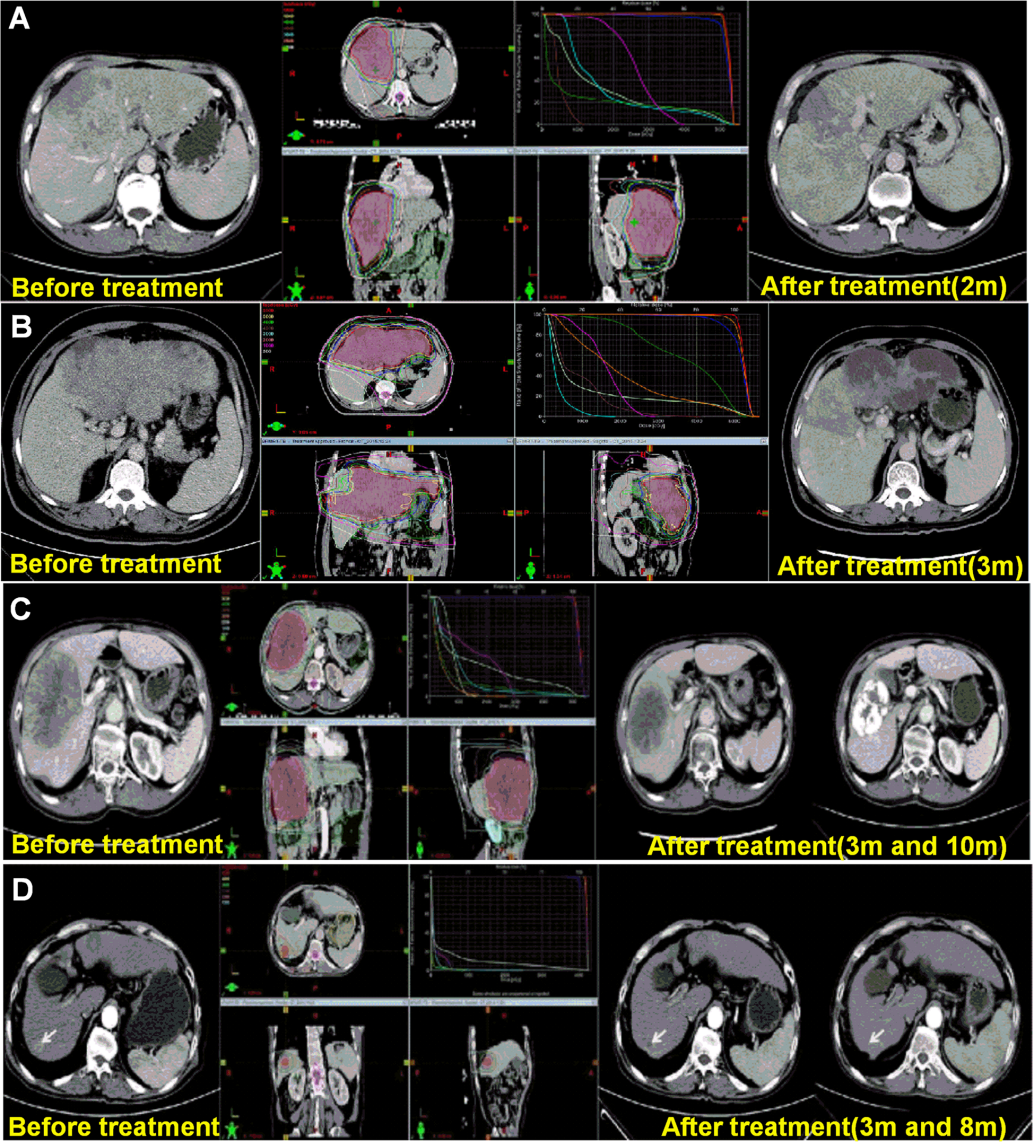
Radiotherapy has a good effect on advanced liver cancer. **a**-**d** Intensity-modulated radiotherapy (IMRT) was used to deliver 5000 cGy to the planning target volume (PTV) in 25 fractions, given once a day over 32 days. **a** CT scans before treatment (*left*) and at 2 months after treatment (*right*). **b** CT scans before treatment (*left*) and at 3 months after treatment (*right*). **c** CT scans before treatment (*left*), at 3 months after treatment, and at 10 months after combination transarterial chemoembolization(TACE) treatment (*right*). **d** CT scans before treatment (*left*) and at 3 months and at 8 months after treatment (*right*)

Primary liver cancer (hepatocellular carcinoma, HCC) is one of the most common malignant cancers worldwide, and represents the third leading cause of cancer-related death [1, 2]. HCC is frequently diagnosed late in its course because of the occult onset and absence of early pathognomonic symptoms. There are often no single effective treatment options available to produce better therapeutic outcomes for poor prognosis [3–5]. Studies have demonstrated that HCC is moderately sensitive to radiation, which is equivalent to the radiosensitivity of low differentiated squamous cell carcinoma [6]. In recent years, with advances in radiation therapy and image processing, such as three-dimensional conformal radiotherapy (3DCRT), stereotactic radiotherapy (SRT), and image-guided radiation therapy (IGRT), radiotherapy has played an increasingly important role in the comprehensive treatment of HCC [4, 7, 8]. Seo et al. performed radiotherapy for 65 patients with advanced unresectable HCC, and noted an objective tumor response rate of 56.9% and a 12-month survival rate of 34.7% [9]^.^ Liang et al. showed that 3-DCRT for 69 cases of massive primary liver cancer (diameter ≥ 10 cm) resulted in a response rate of 47% and a 1-year survival rate of 41% [10]. Onishi et al. reported that for advanced hepatocellular carcinoma with portal vein invasion, the median survival time in the concurrent intra-arterial chemotherapy plus external radiotherapy (CCRT) group was longer than that in the hepatic arterial infusion chemotherapy (HAIC) group (12.4 vs. 5.7 months, respectively) [11]. In recent years, we have treated a certain number of patients (their clinical characteristics are shown in Additional file 1: Table S1) with advanced liver cancer using intensity modulated radiation therapy (IMRT), and have achieved good effects (Fig. 1). However, it is difficult to achieve the tumor radical radiation doses without affecting the many important organs around the lesion, such as normal liver tissue, small intestine, and kidney. Therefore, how to improve the radio-sensitivity of HCC to achieve curative effects in a lower dose range and effectively protect the surrounding vital organs, is an urgent clinical challenge. Thus, identifying novel factors or mechanisms involved in radioresistance might provide scientific evidence for individualized radiotherapy regimens for patients with HCC.

Exposure to a hypoxic environment caused by rapid growth and abnormal vascular system, most cancer cells rely on aerobic glycolysis to generate energy and nutrients to meet their growth needs, instead of mitochondrial oxidative phosphorylation [12, 13]. Therefore, a large amount of lactic acid is produced and accumulated, which leads to decreased sensitivity of cancer cells to radiotherapy [14]. Furthermore, aerobic glycolysis is closely related to tumor radioresistance [15]. Primary HCC is usually accompanied by liver cirrhosis, active proliferation of liver cancer cells, and lack of oxygen. The aerobic glycolysis of liver cancer cells is significantly accelerated [16], and the levels of glycolysis are closely related to cell survival, tumor proliferation, cell apoptosis and invasion ability [17, 18]. For example, Lei et al. showed that FLICE-like inhibitory protein (FLIP_L_) could promote the aerobic glycolysis of HCC cells, which further increased cell tolerance and decreased cell apoptosis in response to low glucose [19]. Xu et al. reported that Chrysin could suppress glycolysis and induce apoptosis in HCC by targeting hexokinase-2 [20]. However, research on whether glycolysis is related to radiotherapy of HCC is still reported rarely at present.

Girdin (also known as ccdc88a, coiled-coil domain containing 88A) is a novel actin binding protein, regulating actin organization and cell motility [21, 22]. Girdin is also involved in the migration of neural stem cells, endothelial cells, and smooth muscle cells, which play important roles in neuron development, angiogenesis, and repair processes after acute myocardial infarction [23–26]. The expression of Girdin is upregulated in multiple malignant tumors, such as colon cancer, glioma, and breast cancer [27–29], promoting carcinogenesis and development, invasion and metastasis, angiogenesis and autophagy, and leading to poor prognosis for patients [24, 25, 27, 29, 30]. Our previous studies suggested that Girdin expression is upregulated in HCC, and correlates closely with tumor size, T stage, TNM stage, and Edmondson–Steiner stage of patients with HCC [31]. We also obtained preliminary data showing that Girdin might accelerate the proliferation, migration, and invasion of HCC cells [31]. However, the involvement of Girdin in glycolysis and radioresistance of HCC has seldom been reported. In the present study, after transfecting a specific small interfering RNA for Girdin into HCC cell lines, HepG2 and Huh-7, glycolysis was significantly inhibited, and radiosensitivity was greatly enhanced compared with control cells. Thus, targeting Girdin and its signal axis will provide new avenues for increasing the sensitivity of HCC to radiotherapy.

## Methods

### Cell culture and transfection

The hepatoma cell lines HepG2 and Huh-7 were purchased from the Type Culture Collection of the Chinese Academy of Sciences, Shanghai, China. Cells were cultured in Dulbecco’s modified Eagle’s medium (DMEM) supplemented with 10% fetal bovine serum (FBS; Gibco, NY), 100 U/mL penicillin, and 100 μg/mL streptomycin, and incubated in a 5% CO_2_ atmosphere at 37 °C. To knockdown Girdin expression, the cells were grown in complete medium for 24 h and then transfected with a short hairpin RNA (shRNA) targeting the *CCDC88A* mRNA (5′-GAAGGAGAGGCAACTGGAT-3′) [27], Girdin-shRNA (Auragene, Changsha, China), by using Lipofectamine™ 2000 (Invitrogen Life Technologies), following the procedure recommended by the manufacturer. After 24 h of transfection, the medium was removed, and the cells were placed in complete medium and maintained at 37 °C in a 5% CO2 incubator.

### Quantitative real-time PCR (qPCR)

To determining mRNA expression of *CCDC88A*, qPCR analysis was performed. Total RNA was isolated using the TRIzol Reagent (Invitrogen Life Technologies) from HepG2 and Huh-7 cells. cDNA was synthesized from 2 μg of total RNA, using random primers with a First Strand cDNA Synthesis Kit (Fermentas, Vilnius, Lithuania). β-actin was used as a housekeeping control. The primer sequences used were: β-actin sense 5′-AGGGGCCGGACTCGTCATACT-3′, antisense 5′-TCCCATGTACCACCACGGCGG-3′; Girdin sense 5′- GACCAACTAGAGGGAACTCG-3′, antisense 5′-TTACCGTGTCTTTGTTTCAT-3′. The real-time PCR was performed with 7300 Fast Real-time PCR system (Applied Biosystems, NY, USA), using the SYBR Green qPCR Mix (TOYOBO). Relative quantification was calculated according to the △△C_T_ method.

### X-ray irradiation

X-ray irradiation was performed using an X-ray generator (Varian, USA), emitting at a fixed dose rate of 4 Gy/min. The energy of the X-rays used to irradiate the cells was graded as 0, 2, 4, 6, and 8 Gy.

### Cell proliferation assay

The HepG2 and Huh-7 cells were incubated in 96-well plates at a density of 5 × 10^3^ cells/well in 100 μL of DMEM and incubated for 24 h after irradiation with graded doses (0, 2, 4, 6, and 8 Gy). Cell proliferation was assessed using an MTT (3-(4,5-Dimethyl-2-thiazolyl)-2,5-diphenyl-2Htetrazolium) assay. Briefly, 10 μL of MTT reagent (Sangon Biotech, China) at 5 mg/mL was added to each well, and the cells were incubated for additional 4 h at 37 °C. Following incubation, the MTT solution was removed and 150 μL of DMSO was added to each well to dissolve purple formazan crystals. After the crystals were completely dissolved, the plate was read at a wavelength of 570 nm using a Microplate Reader (Bio-Rad, USA). Cell proliferation was expressed at a percentage of the relevant controls.

### Cell invasion assay

Cell invasion was measured by Transwell assays. Transfected and irradiated (2 Gy) cells were transferred to the top of EC-Matrigel-coated invasion chambers (Corning, Japan) in 300 μL of serum-free DMEM at a density of 5 × 10^4^ cells/well. Then, 500 μL of DMEM containing 10% FBS was added to the lower chamber. The cells were maintained at 37 °C in a 5% CO_2_ incubator for 24 h. After incubation, non-invading cells were removed using a cotton bud. The invading cells were stained with 500 μL of crystal violet (Beyotime Biotechnology, China) for 20 min. The stained cells were washed by phosphate-buffered saline (PBS) and dried in the air. Images were captured under an inverted microscope (Motic) at 100× magnification. Meanwhile, the crystals were dissolved in 500 μL of glacial acetic acid. Cells were transferred to a 96-well plate to their absorbency at 570 nm was measured using a Microplate Reader (Bio-Rad, USA). Cell invasion was expressed as a percentage of the relevant controls.

### Cell migration assay

Cell migration was determined using a scratch wound healing assay. Transfected and irradiated (2 Gy) cells were seeded in a 6-well plate at a density of 3 × 10^5^ cells/well and cultured to >95% confluence. Wounds of approximately 1 mm in width were scratched vertically into the cell monolayer using a 20-μL sterile pipette tip and the detached cells were removed by aspiration. Medium was replaced with DMEM containing 3% FBS. After incubation for 24 and 48 h, for each group, three fields with a wound area were chosen randomly and photographed under a microscope (Motic). Cell migration was calculated using the following formula: Relative mobility = (distance between the edges of migrated scratches/distance between the edges of initial scratches) × 100%.

### Cell apoptosis assay

Cell apoptosis was evaluated by flow cytometry after staining with an annexinV-fluorescein isothiocyanate (FITC)-propidium iodide (PI) apoptosis detection kit (KeyGEN Biotech, China), according to the manufacturer’s instructions. Fluorescence was measured using a flow cytometer (FACSCanto II, BD). The apoptosis rates were measured by using a FACSCalibur (apoptosis rates = early apoptosis rate + late apoptosis rate), and the data were analyzed with ModFit IT software (Verity Software House, Topsham, ME, USA).

### Colony formation assay

Transfected and irradiated (2 Gy) cells were seeded in soft agar in 10-cm tissue culture dishes and cultured for 1 week to allow colony formation. A colony was defined to consist of at least 10 cells. Colonies were fixed with 4% paraformaldehyde, stained with Giemsa, and counted with the naked eye or using a stereomicroscope. The percentage of colonies formed was expressed as (number of colonies formed/number of cells seeded) × 100%.

### Western blotting

Protein extraction was performed in Radioimmunoprecipitation assay buffer (RIPA) (Auragene, Changsha, China) and centrifuged at 13000 rpm for 20 min at 4 °C. Protein concentrations were determined using a BCA protein assay kit (Auragene, Changsha, China). Proteins were separated on 10% SDS-PAGE gels and then blotted onto nitrocellulose membranes, and probed with Anti-Girdin antibodies (1:1000; ab179481, ABcam); Anti-Glut1 antibodies (1:1000; ab12939, ABcam); Anti-LDHA antibodies (1:2000; ab125683, ABcam); Anti-Hexokinase II antibodies (1:1000; ab76959, ABcam); Anti-HIF1α antibodies (1:2000; ab65069, ABcam); Akt polyclonal antibodies (1:2000; YT0175, Immunoway); Phospho-Akt (T308) polyclonal antibodies (1:2000; YP0007, Immunoway); and Anti-PI3 Kinase p85 antibodies (1:1000; 4292, Cell signaling), followed by the appropriate horseradish peroxidase-conjugated secondary antibodies (Auragene, Changsha, China). Immunodetection was accomplished via the ECL plus western blotting detection system (Auragene, Changsha, China). The signal intensity was determined using the IPP6.0 software. Immunoblotting with anti-β-actin antibodies (1:500) was performed as an internal control.

### Glycolysis analysis

A Glucose Assay Kit, TP Assay Kit, and ATP Assay Kit were purchased from Jiancheng (Nanjing, China); Akt inhibitor IV (Akti IV) was purchased from Cell Signaling Technology (Danvers, MA, USA); PI3K/Akt activator insulin-like growth factor-1 (IGF-1) was purchased from PeproTech (London, UK). The glycolysis process in non-irradiated HepG2 and Huh-7 cells was examined using the Kits above, according to the manufacturer’s instruction. Cell proteins were measured to obtain internal control.

### Animal assay

All animal experiments were approved by the Animal Center of China South University and performed following International Guidelines and Protocols. To generate tumors in vivo, 5 × 10^6^ cells were injected subcutaneously into 4- to 6-week-old BALB/C nude mice on the right flank of their abdomen. When a tumor reached 100 mm^3^ in volume, a single 10 Gy dose of irradiation was delivered. The tumor volume was measured at 2-day intervals. After 41 days, the mouse was sacrificed and the tumors were isolated and measured using vernier calipers. The tumor volume was calculated using the following formula: tumor volume (in mm^3^) = (the longest diameter × the shortest diameter^2^)/2.

### Statistical analysis

Statistical analysis was performed using GraphPrism version 5.0 (GraphPad Software, Inc., La Jolla, CA, USA). Student’s t-test was used to compare quantitative data, and a Chi-square test was used to test qualitative data, such as percentage of cell apoptosis. All values were shown as mean ± SD. *P* < 0.05 was considered to be statistically significant.

## Results

### Silencing of Girdin with shRNA duplexes in HepG2 and Huh-7 cell lines

**Fig. 2:**
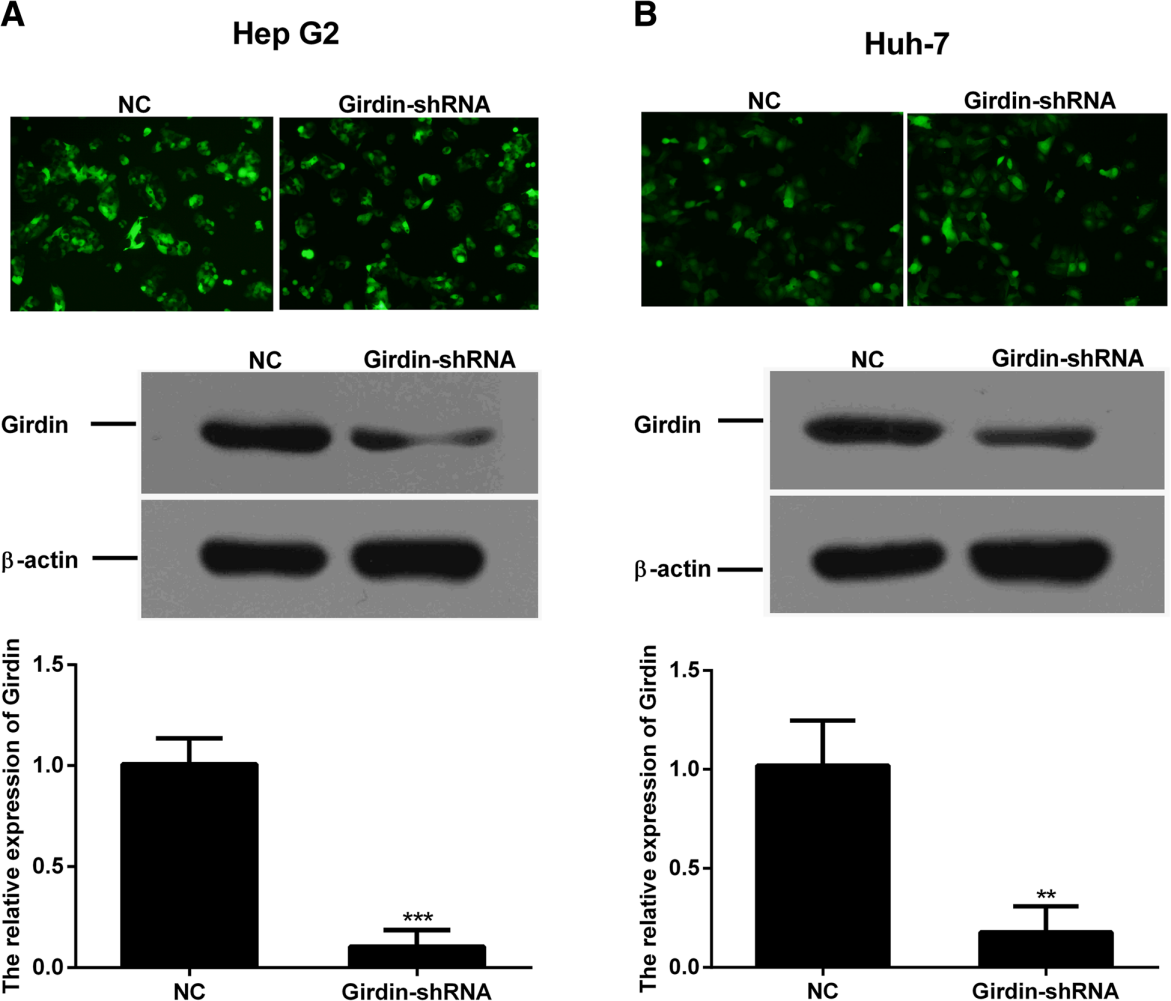
Effect of shRNA on Girdin gene expression in hepatoma cells. **a**, **b** Fluorescence microscopy (×100, upper panel, indicating successful transfection), Protein expression (middle panel), and mRNA expression (*lower panel*) of Girdin in HepG2 (**a**) and Huh-7 cells (**b**). Mean ± SD (*n* = 3 independent experiments). ** *P* < 0.01 and *** *P* < 0.001. NC, normal control; Girdin-shRNA, shRNA transfected cells

Girdin-shRNA was transfected successfully into the hepatic cells HepG2 and Hun-7, and the knockdown effect was verified by qPCR and immunoblotting. The expression of Girdin at the mRNA and protein levels significantly decreased after treatment with the shRNA for 72 h (Fig. 2a, b). This data indicated a successful silencing of *CCDC88A* gene expression induced by the shRNA in hepatocellular carcinoma cells.

### shRNA-mediated silencing of Girdin increases the sensitivity of hepatoma cells to radiation

To examine the influence of Girdin silencing on cellular radiosensitivity, Girdin-shRNA transfected HepG2 and Huh-7 cells were established, and the radiosensitivity of the cells was assessed by cell survival, apoptosis, migration, and invasion assays. Meanwhile, using western blotting to detect the levels of Girdin in HepG2 after irradiation at different doses (0, 2, 4, 6, 8 Gy), we explored the effects of irradiation on Girdin expression; the data showed that X-ray irradiation had no effect on the levels of Girdin (Additional file 1: Figure S1).

**Fig. 3:**
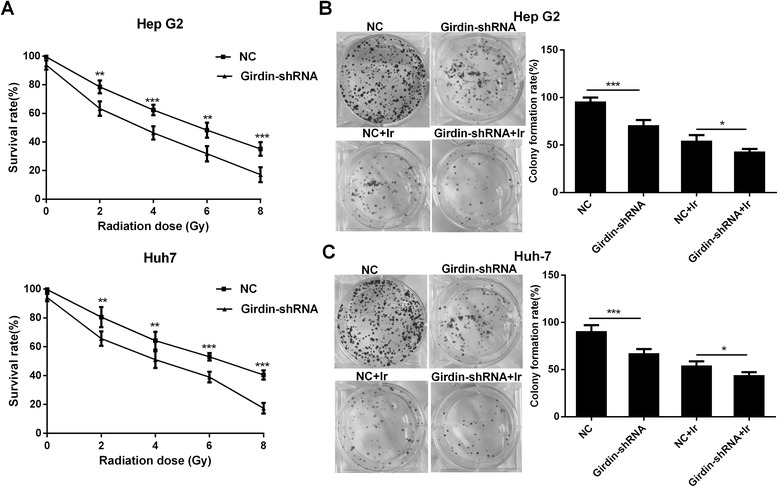
Effects of different treatments on the survival and colony formation ability of hepatoma cells. **a** The cell survival rate of HepG2 and Huh-7 cells transfected with Girdin-shRNA and the corresponding controls. **b**, **c** Colony formation of HepG2 (**b**) and Huh-7 (**c**) cells under different treatments. Mean ± SD (*n* = 3 independent experiments). * *P* < 0.05, ** *P* < 0.01 and *** *P* < 0.001. NC: normal control. Girdin-shRNA, shRNA transfected cells; Ir, 2 Gy of X-rays

Cell survival was measured by the MTT and colony formation assays. In the MTT assay, the survival rate of HepG2 and Huh-7 cells was significantly lower in the Girdin-shRNA transfected group compared with the NC group after irradiation with 2, 4, 6, and 8 Gy of X-rays (Fig. 3a). Similarly, the results from the colony formation assay showed that Girdin-shRNA significantly inhibited colony formation in HepG2 and Huh-7 cells (Fig. 3b, c) after exposure to 2 Gy of X-rays. In addition, basal clonogenic growth without irradiation was also reduced by Girdin knockdown cells (Fig. 3b, c).

**Fig. 4:**
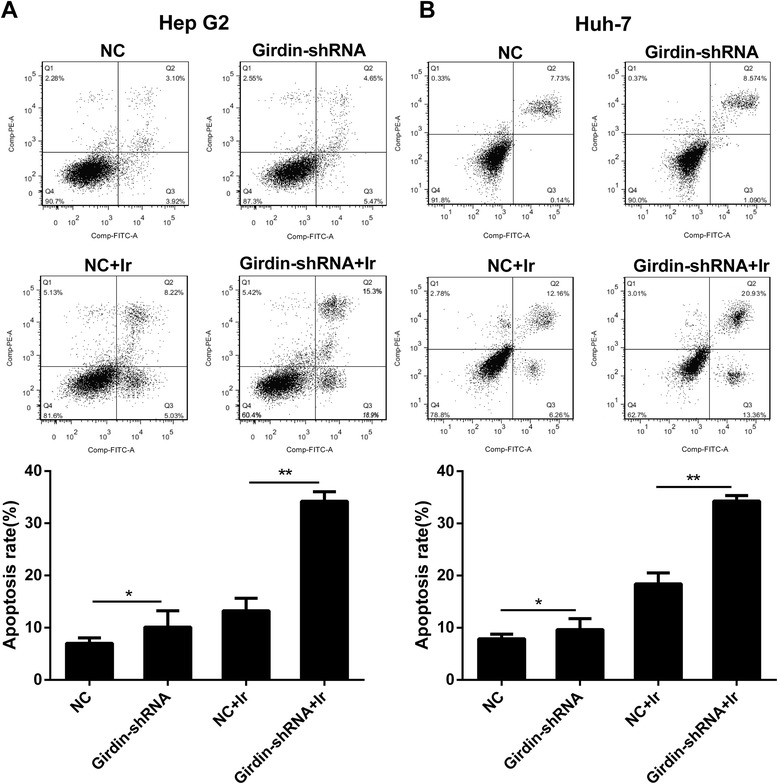
Effects of different treatments on cell apoptosis. **a**, **b** Percentage of apoptosis of HepG2 (**a**) and Huh-7 (**b**) cells under different treatments. Mean ± SD (*n* = 3 independent experiments). * *P* < 0.05 and ** *P* < 0.01 NC, normal control; Girdin-shRNA, shRNA transfected cells; Ir, 2 Gy of X-rays

The radiation (2 Gy)-induced apoptosis was evaluated by a flow cytometry assay. The results demonstrated that, compared with the NC group, the cell apoptosis rate in HepG2 and Huh-7 cells was greatly increased by transfection with the Girdin-shRNA (Fig. 4a, b). In addition, Girdin knockdown promoted basic cell apoptosis in the absence of irradiation (Fig. 4a, b).

**Fig. 5:**
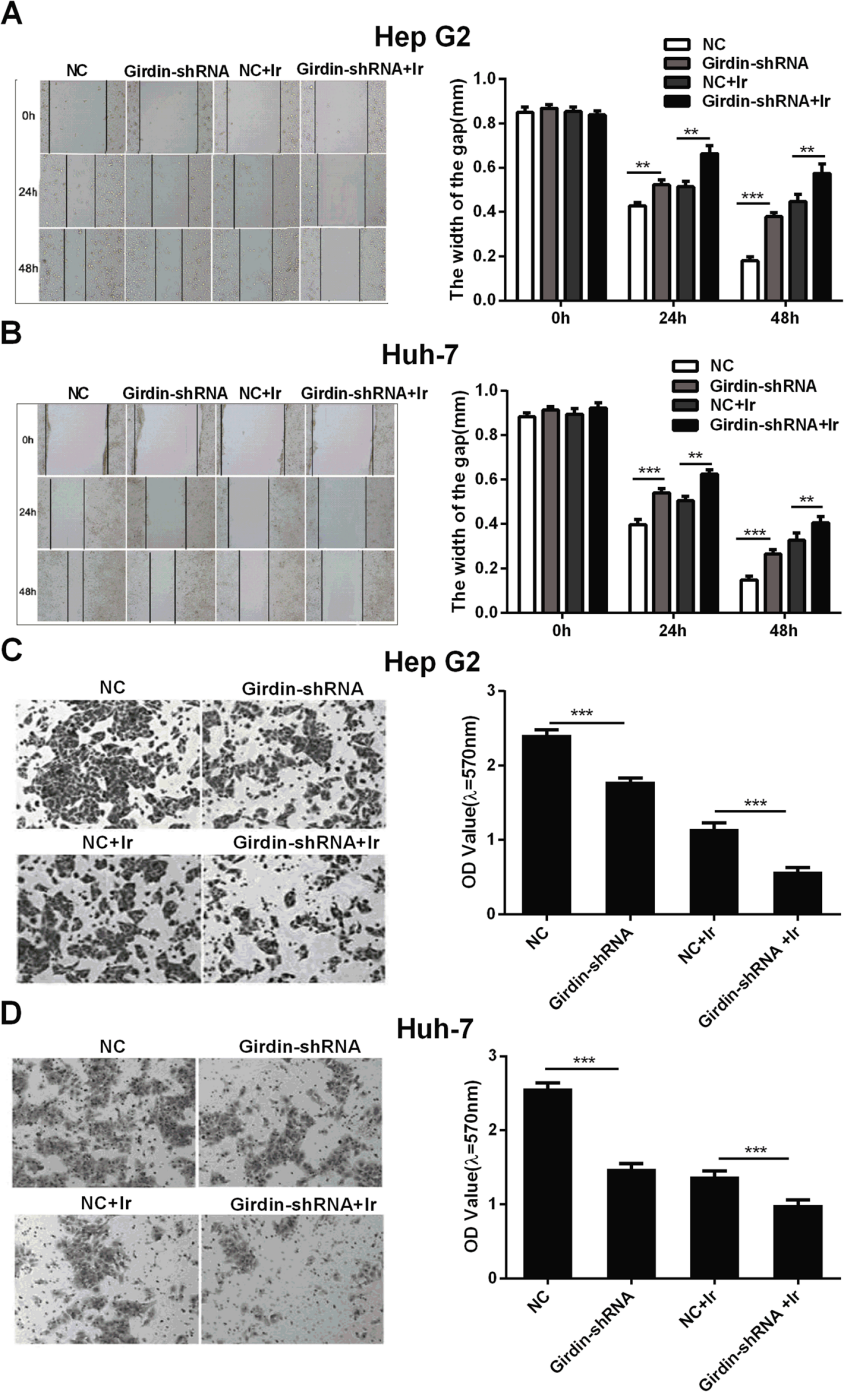
Effects of different treatments on cell migration and invasion. **a**, **b** Scratch wound healing assay showing the migration of HepG2 cells (**a**) and Huh-7 (**b**) cells with different treatments. **c**, **d** Transwell assay showing the invasion of HepG2 cells (**c**) and Huh-7 (**d**) cells with different treatments (original magnification × 200). Mean ± SD (*n* = 3 independent experiments). ** *P* < 0.01 and *** *P* < 0.001. NC, normal control; Girdin-shRNA, shRNA transfected cells; Ir, 2 Gy of X-rays

Cell migration and invasion were determined by scratch and transwell assays, respectively. The scratch wound healing assay showed that the migration of HepG2 and Huh-7 cells treated with Girdin-shRNA was significantly suppressed in the absence or presence of irradiation compared with that in the NC group, as indicated by a decrease in the closed wound area (Fig. 5a, b). Similarly, the transwell assay showed that the Girdin-shRNA inhibited the invasion of HepG2 and Huh-7 cells. These results suggested that knockdown of Girdin could inhibit the invasion of hepatoma cells in vitro (Fig. 5c, d).

Collectively, these data indicated that Girdin-shRNA transfected hepatoma cells showed significantly increased radiosensitivity compared with normal hepatoma cells after irradiation exposure.

### Girdin-shRNA treatment suppresses glycolysis of hepatoma cells

**Fig. 6:**
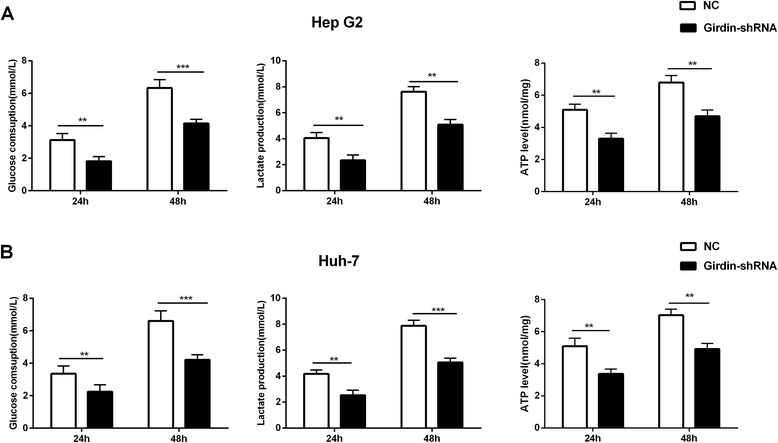
Girdin knockdown inhibits glycolysis in hepatoma cells. **a**, **b** Effect of the Girdin-shRNA on glucose uptake, and lactate and ATP production, at 24 and 48 h after transfection of HepG2 (**a**) and Huh-7 cells (**b**). Mean ± SD (*n* = 3 independent experiments). **, *P* < 0.01 and ***, *P* < 0.001. NC, normal control; Girdin-shRNA, shRNA transfected cells

To identify the molecular mechanism of the enhanced sensitivity after irradiation in Girdin-shRNA transfected cells, we performed a glycolysis assay. Our data showed that silencing of Girdin strongly inhibited the ability of HepG2 and Huh-7 cells to take up glucose and produce lactate and ATP (Fig. 6a, b).

### Girdin-shRNA-induced suppression of glycolysis is mediated by the PI3K-AKT pathway

**Fig. 7:**
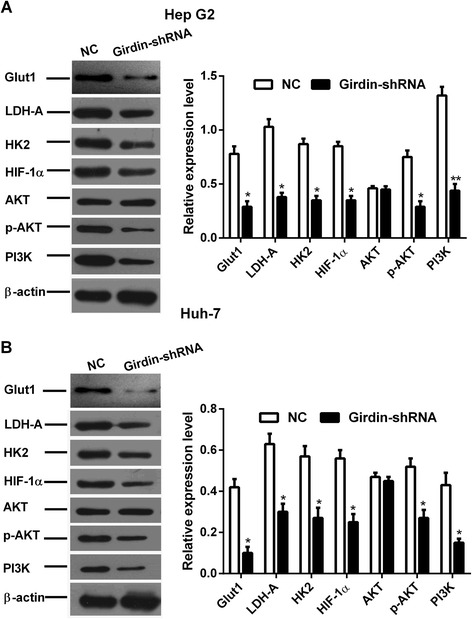
Girdin knockdown inhibits the expression of glycolysis-related proteins and the PI3K-Akt signaling pathway. **a**, **b** Immunoblots showing the levels of Glut1, LDH-A, HK2, HIF-1α, AKT, p-AKT, and PI3K in HepG2 (**a**) and Huh-7 cells (**b**), with or without Girdin-shRNA treatment, but without radiation. Mean ± SD (*n* = 3 independent experiments). *, *P* < 0.05 and **, *P* < 0.01. NC, normal control; Girdin-shRNA, shRNA transfected cells; Glut1, Glucose transporter 1; LDH-A, Lactic dehydrogenase-A; HK2, Hexokinase 2; HIF-1α, Hypoxia inducible factor 1α; AKT. Protein-serine-threonine kinases; p-AKT, phosphorylated AKT; PI3K, phosphatidylinositol 3-kinase

We further explored the signaling pathway downstream of Girdin. Western blotting experiments showed that PI3K levels were downregulated, and AKT phosphorylation was further inhibited in HepG2 and Huh-7 cells transfected with Girdin-shRNA compared with that in the NC group. In addition, the levels of HIF-1α and glycolysis-related proteins, Glut1, LDHA, and HK2, were decreased in Girdin-shRNA treated cells (Fig. 7a, b).

**Fig. 8:**
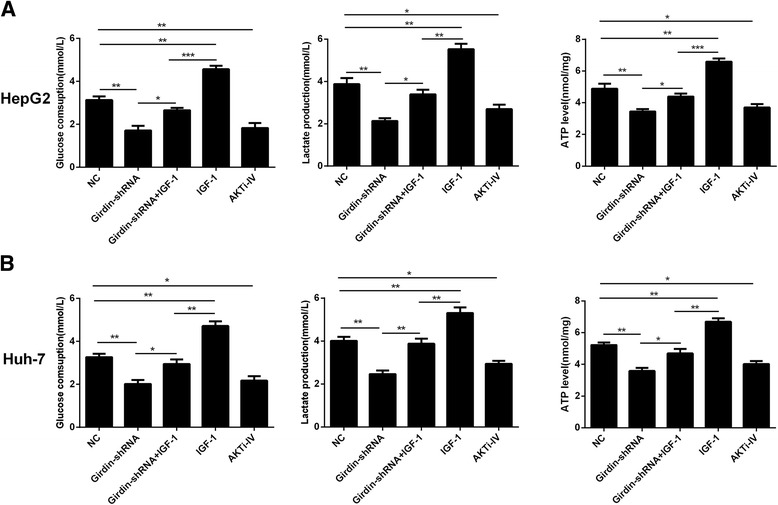
The PI3K/AKT signaling pathway regulates the process of glycolysis in hepatoma cells. **a**, **b** Effects of Girdin-shRNA, IGF-1, and Akti IV on glucose uptake, and lactate and ATP production, at 24 h after with different treatments in HepG2 (**a**) and Huh-7 cells (**b**). Mean ± SD (*n* = 3 independent experiments). *, *P* < 0.05, **, *P* < 0.01, and ***, *P* < 0.001. NC, normal control; Girdin-shRNA, shRNA transfected cells; IGF-1, insulin-like growth factor-1; Akti IV, Akt inhibitor IV

Furthermore, we investigated if PI3K/Akt played a role in the process of glycolysis of hepatoma cells using the PI3K/Akt activator insulin like growth factor 1 (IGF-1) and the AKT inhibitor Akti IV. IGF-1 is a well-known PI3K signaling activator that plays an important role in the regulation of cell proliferation and differentiation. Treatment with IGF-1 resulted in a significant increase in the level of glycolysis compared with NC, and reactivating the PI3K/Akt pathway with IGF-1 partly reversed the shRNA-induced downregulation of cellular glycolysis levels (Fig. 8a, b). Akti IV prevents ATP binding by a kinase upstream of Akt, but downstream of PI3K. Treatment with Akti IV resulted in significant inhibition of glycolysis of hepatoma cells compared with NC (Fig. 8a, b). Taken together with these results, knockdown of Girdin expression suppressed the glycolysis of hepatoma cells by inhibiting the PI3K-Akt signaling pathway.

### Silencing of Girdin expression enhances the radiosensitivity of heptoma cells by negatively regulating glycolysis in vivo

**Fig. 9:**
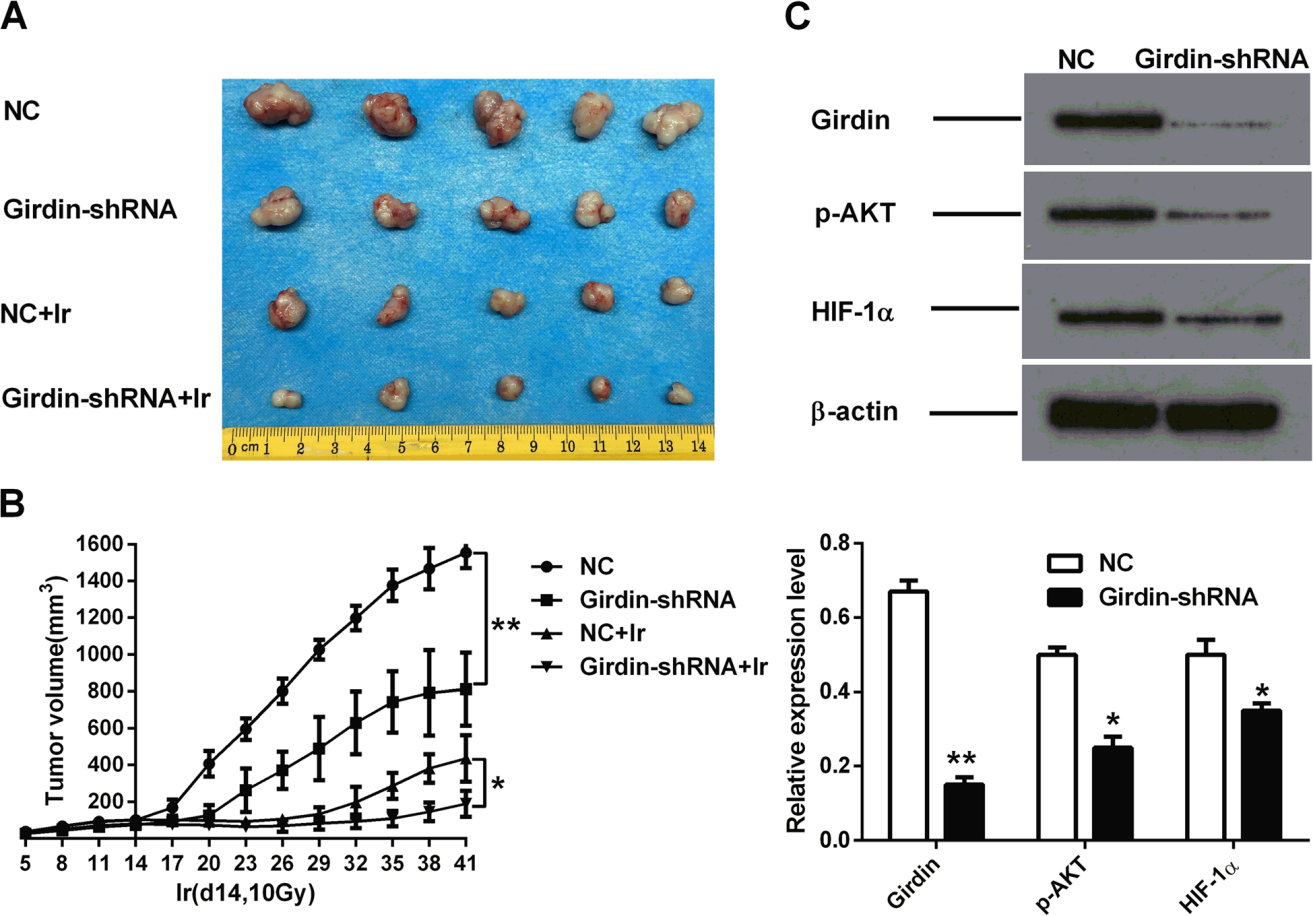
Silencing Girdin increases the radiosensitivity of hepatoma cells in vivo. **a** Photograph of tumors derived from Girdin-shRNA transfected or untransfected HepG2 cells at the end of the observation period. Tumor tissues were irradiated or not at 2 weeks. **b** Tumor growth curve in nude mice. After tumor cells were injected subcutaneously into the right flank of the abdomen of nude mice, the short and long diameters of the tumors were measured every 3 days and tumor volumes (mm^3^) were calculated. Mean ± SD (*n* = 5/group). **c** Western blotting of Girdin, p-AKT, and HIF-1α protein expression in HepG2 xenografts.*, *P* < 0.05 and **, *P* < 0.01. NC, normal control; Girdin-shRNA, shRNA transfected cells; Ir, 10 Gy of X-rays

The effect of Girdin silencing on the radiosensitivity of hepatoma cells in vivo was investigated by injecting Girdin-shRNA transfected HepG2 cells and control cells subcutaneously into nude mice. Two weeks after tumor inoculation, murine tumor tissues were exposed to a 10 Gy single-dose of X-ray. Tumor sizes were measured every 3 days. As a result, the tumor volumes in the Girdin-silenced group were smaller than those in their corresponding control groups, with or without irradiation (Fig. 9a, b). To analyze the relationship between radiosensitivity and glycolysis, western blotting analysis was performed to measure the Girdin, p-AKT, and HIF-1α protein levels in the xenograft tumors (Fig. 9c). The results showed that Girdin, p-AKT, and HIF-1α proteins were significantly downregulated after knockdown of Girdin. Therefore, the in vivo experiments further confirmed that silencing of Girdin expression in HCC cells increased radiosensitivity, accompanied by downregulation of glycolysis.

## Discussion

It is reported that the expression of Girdin is elevated in many malignant tumorous tissues, such as liver cancer, breast cancer, and colon cancer. Its ectopic expression promotes tumor migration and invasion, and is closely related to poor prognosis and drug resistance [27, 29, 31]. However, the relationship between Girdin expression and tumor radiotherapy has not been clear until now. In the present study, we found the knockdown of Girdin expression in HCC cells significantly inhibited cell proliferation, colony formation, tumor metastatic invasion, and growth, and promoted cell apoptosis. These results are consistent with our previous studies. Furthermore, after exposure to X-rays, the invasion and metastasis of Girdin-silenced cells was significantly reduced compared with the control group. In addition, the radiation-induced apoptosis of Girdin silenced cells was increased obviously. Tumor growth in vivo was also inhibited by the Girdin shRNA. Taken together, these results suggested that shRNA mediated-silencing of Girdin expression could enhance the sensitivity of HCC to radiotherapy. We then demonstrated that glucose uptake and the production of lactate and ATP were decreased in Girdin shRNA-treated cells, which indicated that Girdin might promote glycolysis in cancer cells. Finally, we also showed that after Girdin silencing, the levels of glycolysis-related signaling factors, PI3K, p-AKT and HIF-1α, and enzymes, Glut1, LDH-A and HK2, were downregulated. Simultaneously, the Girdin shRNA-induced reduction in cell glycolysis was partly reversed by IGF-1. These data suggested that Girdin might regulate glycolysis, which affects the radiosensitivity of HCC via the PI3K-AKT-HIF1α signaling pathway.

The metabolism of tumor cells differs from that of normal cells. In particular, altered energy metabolism is a new feature of malignant tumors. The glucose metabolism of tumor cells is mainly characterized by an increase in glucose uptake and lactate production, a phenomenon termed “the Warburg effect” [12, 32]. The lack of oxygen in the tumor microenvironment means that tumor cells rely on increasing glycolysis to meet the demand for energy. In the present study, we found the knockdown of Girdin expression by an shRNA in HCC cells resulted in decreased glucose uptake and ATP production, suggesting that Girdin might regulate energy metabolism in the process of tumorigenesis and development. The antioxidative capacity of lactate might contribute to radioresistance in malignant tumors, depending on its clearance of superoxide and hydroxyl radicals, which can damage the tumor DNA [14, 33]. Our data showed that the production of lactate in HCC cells was significantly decreased after silencing Girdin expression, which might contribute to the increased radiosensitivity of HCC cells. Taken together, our results indicated that shRNA-induced Girdin silencing enhances the sensitivity of HCC cells to radiation via inhibiting glycolic metabolism.

Girdin binds to Giα3 and then stimulates the PI3K/AKT signaling pathway, which participates in the proliferation, invasion, and metastasis of malignant tumors [34, 35]. HIF-1α is a hypoxia-induced transcription factor whose expression is regulated by PI3K/AKT signaling pathway. After stimulation, HIF-1α translocates to the nucleus and its transcriptional activity is increased [36]. HIF-1α then upregulates the expression of glycolysis-related proteins, which accelerates glucose metabolism to satisfy the requirement for energy [37, 38]. Many studies have proved that HIF-1α plays an important role in increasing radioresistance of cancer cells via the regulation of glucose metabolism, the cell cycle, and radiation-induced cell apoptosis [39–42].

With the intensive development of radiotherapy for cancer, an increasing number of studies have investigated how to improve the efficacy of this treatment option. The challenges of tumor radioresistance resulting from hypoxia-induced glycolysis should be resolved urgently. Our findings demonstrated that Girdin could regulate the glycolysis of HCC cells through the PI3K/AKT/HIF-1α signaling pathway, which affects the sensitivity of tumor cells to radiotherapy. Therefore, targeting Girdin and its related signal axis is a novel and promising treatment strategy to enhance the radiosensitivity of HCC.

## Conclusions

In summary, we demonstrated that Girdin could regulate the glycolysis of HCC cells via the PI3K/AKT/HIF-1α signaling pathway, which affects the sensitivity of tumor cells to radiotherapy. Our findings suggested that targeting Girdin and its signal axis would provide new avenues to increase the sensitivity of HCC to radiotherapy.

## Additional file

Additional file 1:
**Table S1.** Clinical characteristics of the 22 HCC patients with IMRT. **Figure S1.** Effect of X-ray irradiation on Girdin gene expression in hepatoma cells. (DOCX 122 kb)

## Abbreviations

AKT: Protein-serine-threonine kinase
Girdin-shRNA: shRNA transfected cells
Glut1: Glucose transporter 1
HCC: Primary hepatocellular carcinoma
HIF-1α: Hypoxia inducible factor 1α
HK2: Hexokinase 2
IMRT: Intensity modulated radiation therapy
LDH-A: Lactic dehydrogenase-A
NC: Normal control
p-AKT: Phosphorylated AKT
PI3K: Phosphatidylinositol 3-kinase
