# Supplementary material for: Silencing the Girdin gene enhances radio-sensitivity of hepatocellular carcinoma via suppression of glycolytic metabolism
Source: J Exp Clin Cancer Res. 2017 Aug 15;36:110. doi: 10.1186/s13046-017-0580-7 (PMC5558715; doi:10.1186/s13046-017-0580-7)
Supplement: Additional file 1: — Table S1. Clinical characteristics of the 22 HCC patients with IMRT. Figure S1. Effect of X-ray irradiation on Girdin gene expression in hepatoma cells. (DOCX 122 kb) [file 13046_2017_580_MOESM1_ESM.docx]

**Table S1 Basic clinical data for 22 HCC patients with IMRT**

| **Name** | **Gender** | **Age (years)** | **General type** | **Histological type** | **effects** |
| --- | --- | --- | --- | --- | --- |
| Xsl | Female | 43 | Giant liver cancer | Hepatocellular carcinoma | PR |
| Lsd | Male | 35 | Giant liver cancer | Hepatocellular carcinoma | PR |
| Xl | Female | 55 | Giant liver cancer | Cholangiocarcinoma | SD |
| Qdq | Female | 56 | Giant liver cancer | Hepatocellular carcinoma | PD |
| Ljl | Male | 45 | Small liver cancer | Hepatocellular carcinoma | SD |
| Hyz | Female | 60 | Small liver cancer | Hepatocellular carcinoma | PR |
| Yxj | Male | 59 | Small liver cancer | Cholangiocarcinoma | PR |
| Tfs | Male | 41 | Small liver cancer | Hepatocellular carcinoma | PR |
| Zyj | Female | 56 | Small liver cancer | Hepatocellular carcinoma | PR |
| Pgh | Male | 50 | Giant liver cancer | Hepatocellular carcinoma | SD |
| Gpl | Female | 57 | Giant liver cancer | Hepatocellular carcinoma | PD |
| Fld | Male | 45 | Small liver cancer | Hepatocellular carcinoma | CR |
| Mcm | Male | 48 | Small liver cancer | Hepatocellular carcinoma | PR |
| Rbl | Male | 52 | Small liver cancer | Cholangiocarcinoma | PR |
| Zkl | Male | 49 | Small liver cancer | Cholangiocarcinoma | SD |
| Wxx | Female | 52 | Giant liver cancer | Hepatocellular carcinoma | PR |
| Wrp | Male | 52 | Small liver cancer | Hepatocellular carcinoma | CR |
| Hgh | Male | 57 | Giant liver cancer | Hepatocellular carcinoma | PR |
| Gxy | Female | 48 | Small liver cancer | Hepatocellular carcinoma | PR |
| Ycy | Female | 51 | Small liver cancer | Hepatocellular carcinoma | PR |
| Qcq | Male | 61 | Giant liver cancer | Cholangiocarcinoma | SD |
| Zxj | Male | 54 | Giant liver cancer | Hepatocellular carcinoma | PD |





**Figure S1 Effect of X-ray irradiation on Girdin gene expression in hepatoma cells.**

Western blot analysis of Girdin expression in HepG2 cell treated with X-ray irradiation. The cell was exposed to different dose of radiation (0, 2, 4, 6, 8 Gy) for 24 hours. Mean ± SD (n=3 independent experiments).
